# Role of fomites in SARS transmission during the largest hospital outbreak in Hong Kong

**DOI:** 10.1371/journal.pone.0181558

**Published:** 2017-07-20

**Authors:** Shenglan Xiao, Yuguo Li, Tze-wai Wong, David S. C. Hui

**Affiliations:** 1 Department of Mechanical Engineering, The University of Hong Kong, Hong Kong SAR, China; 2 JC School of Public Health and Primary Care, The Chinese University of Hong Kong, Prince of Wales Hospital, Shatin, Hong Kong SAR, China; 3 Department of Medicine and Therapeutics, The Chinese University of Hong Kong, Prince of Wales Hospital, Shatin, Hong Kong SAR, China; Columbia University, UNITED STATES

## Abstract

The epidemic of severe acute respiratory syndrome (SARS) had a significant effect on global society in the early 2000s and the potential of its resurgence exists. Studies on the modes of transmission of SARS are limited though a number of outbreak studies have revealed the possible airborne route. To develop more specific and effective control strategies, we conducted a detailed mechanism-based investigation that explored the role of fomite transmission in the well-known Ward 8A outbreak. We considered three hypothetical transmission routes, i.e., the long-range airborne, fomite and combined routes, in 1,744 scenarios with combinations of some important parameters. A multi-agent model was used to predict the infection risk distributions of the three hypothetical routes. Model selection was carried out for different scenarios to compare the distributions of infection risk with that of the reported attack rates and select the hypotheses with the best fitness. Our results reveal that under the assumed conditions, the SARS coronavirus was most possible to have spread via the combined long-range airborne and fomite routes, and that the fomite route played a non-negligible role in the transmission.

## Introduction

The severe acute respiratory syndrome coronavirus (SARS-CoV) was a substantial global threat associated with significant morbidity and mortality in the early 2000s [1]. Since its emergence in November 2002, the SARS-CoV had induced 8,096 cases, including 774 deaths, in 37 countries within 8 months [2]. Although no new outbreaks have been reported since 2004 [3], reported biosecurity breaches of SARS-CoV specimens in research facilities [4–7] and continuous findings of SARS-like coronaviruses in wild animals [8–10] suggest the distinct potential for a resurgence of SARS [11–13].

**Fig 1:**
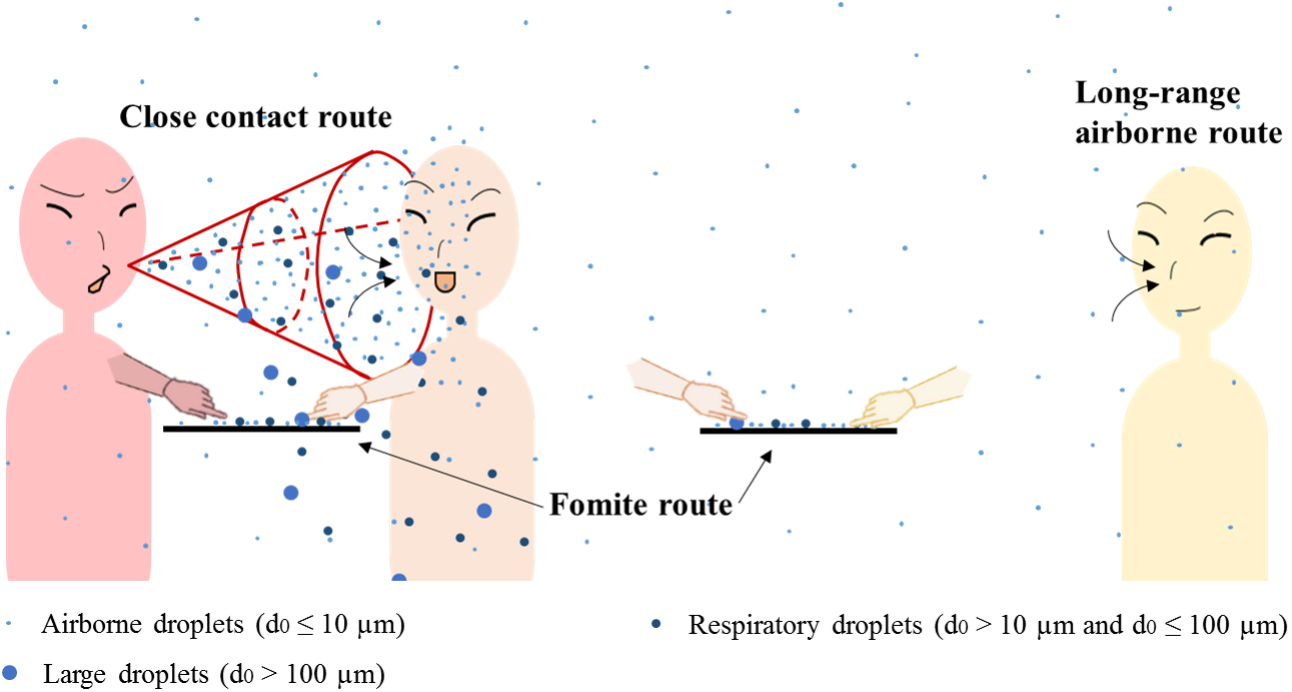
The three major transmission routes: Long-range airborne, close contact and fomite. The person in red is the index patient.

Like many other respiratory viruses, the SARS-CoV is suspected to spread from an infected person to the susceptible via three basic transmission routes, i.e., the long-range airborne, close contact and fomite routes [14–16], as shown in Fig 1. Understanding of the relative importance of the three routes is limited, so the recommended infection control measures (standard, contact, droplet and airborne precautions [12, 17]) have been vague and unfocused. Due to safety and ethical concerns, experiments on human subjects are not appropriate [18]. Several studies have proposed probable evidence for the airborne spread of the SARS-CoV based on the consistencies between bio-aerosol concentration distributions and reported attack rates [19–21], but no mechanism-based investigations exist for the fomite route. Nevertheless, the detection of positive environmental samples in SARS outbreak hospitals [22–24], infections caused by intranasal instillation in animal experiments [25, 26] and findings that hand washing reduces the infection rate [27–29] all reveal that the fomite route might have played a non-negligible role in transmission.

**Fig 2:**
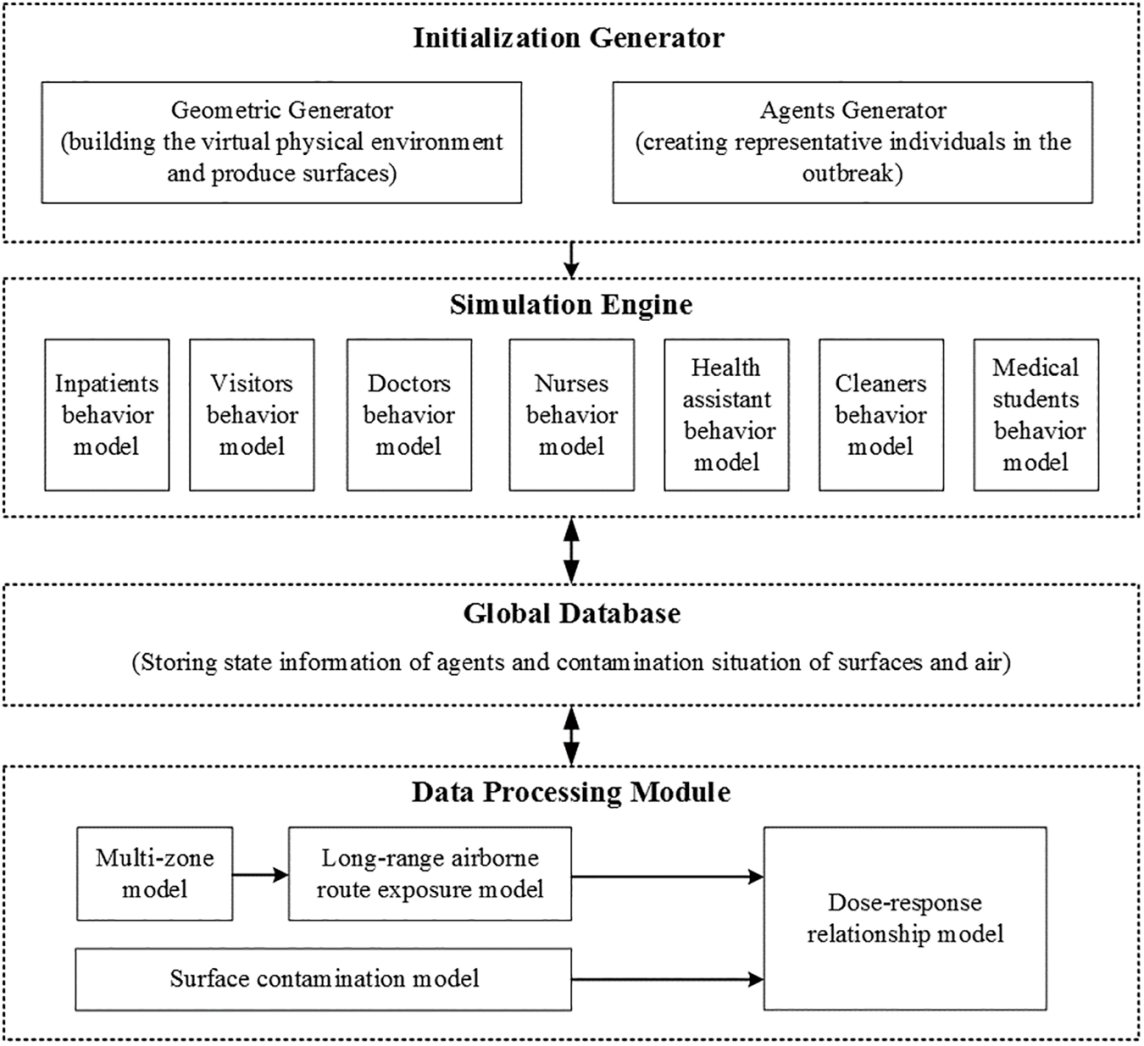
System architecture of the multi-agent model.

To investigate the role the fomite route plays in SARS-CoV transmission, we conducted a detailed modelling study of the largest hospital outbreak in Hong Kong [20], in which the distribution of reported attack rates of inpatients showed a statistically significant spatial pattern. Since the index inpatient was weak and bedridden [30], we excluded the possibility of the close contact route from the index patient to other inpatients and identified three hypotheses, namely the single-route long-range airborne transmission (Hypothesis 1 [Long Air]), the single-route fomite transmission (Hypothesis 2 [Fomite]) and the two-route combination (Hypothesis 3 [Long Air + Fomite]). Based on a typical 3-shift rotation over 24 hours, six routine round patterns of healthcare workers (HCWs) were considered. A multi-agent model (Fig 2) was developed to simulate the possible spread of the viruses from the index patient to the susceptible by air flow and surface touching, and to calculate the possible exposure doses and infection risks for each hypothesis. Model selection was carried out in 1,744 scenarios with various combinations of 4 important parameters. The results reported as follows provide probable evidence for the additional fomite transmission of the SARS-CoV under assumed conditions.

## Methods

### The outbreak

**Fig 3:**
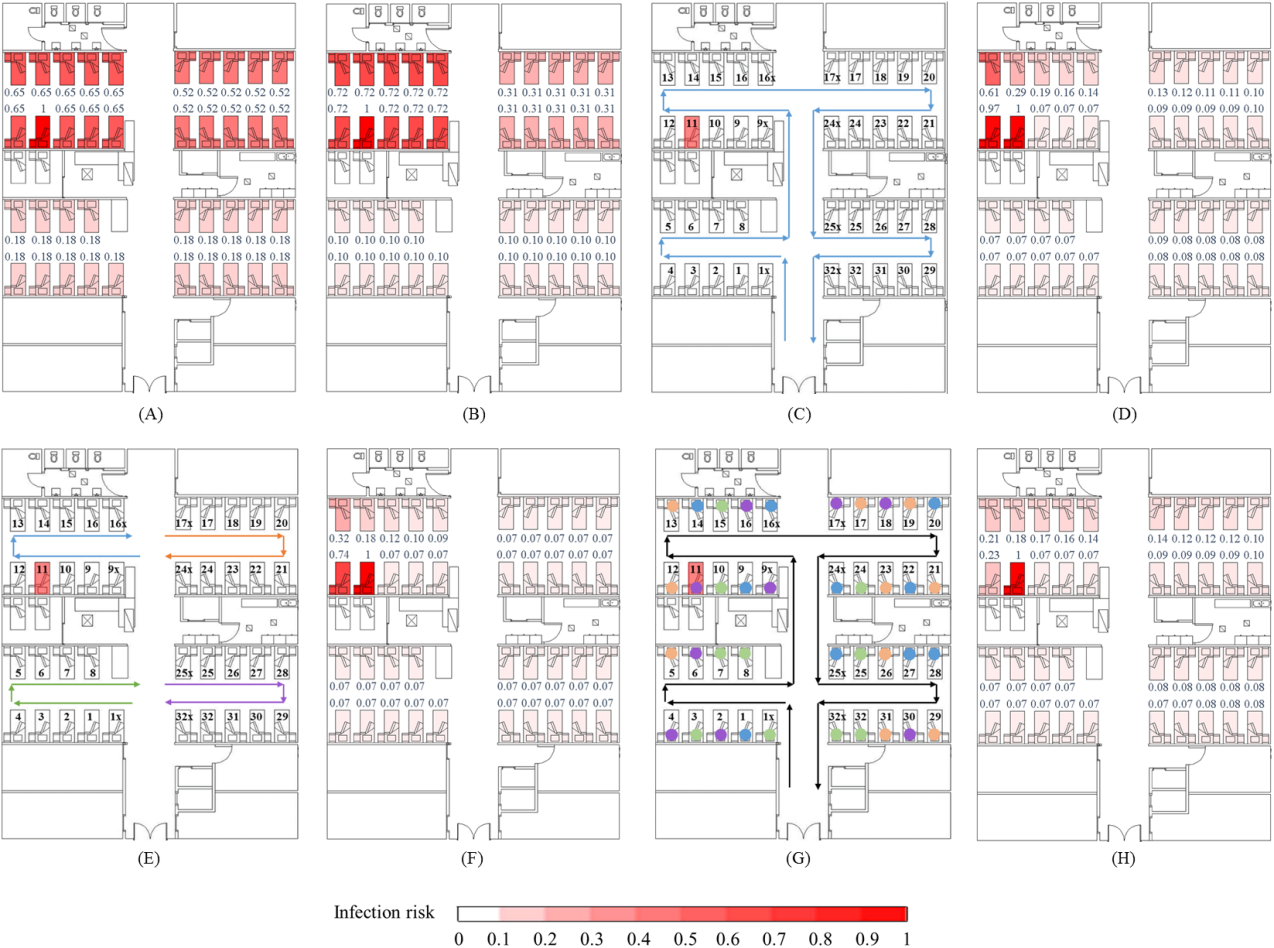
Distributions of reported attack rates and predicted infection risk and HCWs routine round patterns. (A) Reported attack rates distribution [20]. (B) Predicted average infection risk distribution (for 1,000 simulations) via the long-range airborne route at 24:00 on March 12, the end of the exposure period. (C) HCWs routine round Pattern 1. (D) Predicted average infection risk distribution via the fomite route (Pattern 1). (E) HCWs routine round Pattern 3. (F) Predicted average infection risk distribution via the fomite route (Pattern 3). (G) HCWs routine round Pattern 5. (H) Predicted average infection risk distribution via the fomite route (Pattern 5). The largest virus-containing droplet size *d*_*g*_ = 100 μm, dose–response parameters in respiratory tracts *η*_*r*_ = 3.2/mRNA copy and on mucous membranes *η*_*m*_ = 3.2 × 10^−3^/mRNA copy and the viral load coefficient *c*_*L*_ = 10. Bed numbers are marked in black in (C), (E) and (G). Reported attack rates and predicted average infection risks for every inpatient are marked in blue in (A), (B), (D), (F) and (H). The intensity of red shading represents levels of attack rate or infection risk.

As shown in Fig 3A, the outbreak occurred in a general medical ward, Ward 8A, in the Prince of Wales Hospital in early March 2003 [31]. The index patient was a 26-year-old man who developed fever and cough on February 24, 2003 and was admitted to Bed 11 in Ward 8A on March 4 [32]. As his condition deteriorated with difficulty in expectorating sputum, he was treated with salbutamol via a jet nebulizer four times a day from March 6 to March 12 to facilitate mucociliary clearance [20]. On March 13, 2003, after he was identified as the index patient for the outbreak, he was transferred to an isolation room [20]. Thus, the period of March 4–12, 2003 was taken as the suspected exposure period.

Seven groups of people are assumed to be involved in transmission during the exposure period, including inpatients, visitors, doctors, nurses, health assistants, cleaners and medical students. The infection patterns of inpatients were studied because their behaviour was simpler than that of HCWs and visitors, and they presented more available data than medical students [20]. During the hospitalisation of the index patient, 30 of 74 inpatients were infected. As shown in Fig 3A, the distribution of infected inpatients exhibited a clear pattern (*P* = 0.0015, Pearson chi-square test), with the highest attack rate (0.6500, 13 of 20 inpatients) in the source cubicle (including Beds 9x, 9–16 and 16x), a little lower (0.5238, 11 of 21 inpatients) in the adjacent cubicle (including Beds 17x, 17–24 and 24x) and lowest (0.1818, 6 of 33 inpatients) in the remote cubicles (including Beds 1x, 1–8, 25x, 25–32 and 32x) [20].

### Major assumptions

As the information for this outbreak and the studies on properties of SARS-CoV are not sufficient, we made three major assumptions to build our model as follows. First, six representative HCWs’ routine round patterns were considered, and the contact modes between HCWs and different patients were assumed to be the same. As shown in Fig 3C and Figure D(iii) in S1 File, doctors and nurses were responsible for all of the inpatients, and they examined all of the patients in the ward in clockwise and anticlockwise directions in Patterns 1 and 2, respectively. As shown in Fig 3E and Figure D(v) in S1 File, each doctor and each nurse was responsible for inpatients in a cubicle, and examined them in clockwise and anticlockwise directions in Patterns 3 and 4, respectively. In addition, in the scenario in which doctors or nurses allocated the patients in the order in which they checked in, the inpatients for whom a doctor or a nurse took responsibility might have been random. In that scenario, each doctor and each nurse examined a random nine or ten inpatients (e.g., circles of four colours in Fig 3G and Figure D(vii) in S1 File) in the ward in clockwise and anticlockwise directions in Patterns 5 and 6, respectively.

Second, the uncertain parameters related to the properties of SARS-CoV were assumed and individual differences were not considered, as listed in S1 File. These parameters included surface areas (Table B in S1 File), transfer rate between surfaces (Table C in S1 File), virus inactivation rates on surfaces (Table D in S1 File), virus loads (Table E in S1 File), dose-response parameters (Table F in S1 File) and the largest virus-containing droplet size (Table I in S1 File).

Third, for all susceptible patients, the length of exposure period were assumed to be same (March 4–12, 2003) and the index patient was assumed to be the only source. Since the information about admission and discharge timing of patients were vague in the outbreak related reports and researches [20, 30, 32], we could not estimate the exposure period for every patient. As for the virus source, although there were 13 normal patients getting infected during the exposure period [20], the viral load was still low compared to the index patient [33]. Therefore, we did not consider the transmission from early-onset cases to the later cases.

### The multi-agent modelling framework

A multi-agent model was used to model the spread of the SARS-CoV from the index patient to the susceptible and predict the infection risk distributions from the three hypotheses. Fig 2 shows the system architecture of the modular-based model, which includes four parts: the initialization generator, simulation engine, global database and data processing module.

Initialization Generator had two branches, namely Geometric Generator and Agents Generator. Geometric Generator was used to build the virtual physical environment and produce surfaces. Eighteen kinds of representative surfaces were identified (Table A in S1 File) and categorised into five types of material: porous surfaces, non-porous surfaces, toilet surfaces, skin and mucous membranes, which differed in their properties (Tables B and C in S1 File). In this study, ‘mucous membranes’ refers in particular to the exposure site for the fomite route, namely the mucous membranes of eyes, noses and mouths [15]. Agents Generator was used to create representative individuals in the outbreak. Agents in seven representative roles (inpatients, visitors, doctors, nurses, health assistants, cleaners and medical students) were identified as study objects, (Table H in S1 File) and each agent corresponded to a person in the outbreak.

The core of the model, the simulation engine, including seven behaviour models, was used to set behaviour rules and simulate the behaviour of agents. The frequencies and the touching sequences for different types of behaviour are shown in Tables F and G in S1 File. The heterogeneity was retained for every agent, so agents behaved independently. After every time step, the information about the agents was sent to update the global database, which temporarily recorded the agents’ state information and the contamination situations of surfaces and air.

The data processing module was used to calculate the exposure dose and infection risk. For the long-range airborne route, the multi-zone model [21, 34, 35] and long-range airborne route exposure model [36] were used to acquire the aerosol concentrations in the six zones of Ward 8A and exposure doses in the respiratory tract, respectively. For the fomite route, a surface contamination model was used to calculate the number of viruses exchanged between surfaces in every touching process and the exposure doses on the mucous membranes. The infection risk of every agent for the three hypothesised transmission modes was calculated by the dose–response relationship model [36, 37]. Details of these mathematical models are provided in S1 File.

### Model selection

With the multi-agent modelling framework, we calculated the average infection risk for every region (source cubicle, adjacent cubicle and remote cubicles). In this study, maximizing fit was selected as the approach to model selection [38]. In this approach, the residual sum of squares (RSS), as a measure of fit [39], was calculated for every hypothesis. Since a small RSS indicates a good fit of the model to the data, the hypothesis with the minimum RSS was selected.

In this study, since several uncertain parameters related to the properties of SARS-CoV were assumed, we investigated some important ones and discussed their value ranges. As suggested by Gao [36], the largest virus-containing droplet size, dose–response parameters in respiratory tracts and on mucous membranes and viral load all greatly influence infection risk, but the related measurements are lacking in the literature. In this study, the viral loads during the exposure period (March 4–12, 2003) were assumed to vary according to the measured data of Peiris et al. [33], increasing at first, reaching a peak on the 10th day after the onset of symptoms and then decreasing. Therefore, the viral load coefficient was defined as the ratio of the viral load in the computation to the average values in [33]. The coefficient was assumed to be constant for the total exposure period. To reduce the number of variables, *η*_*r*_, *η*_*m*_ and *c*_*L*_ were combined as the products *η*_*r*_*c*_*L*_ and *η*_*m*_*c*_*L*_, defined as the dose effects of introducing *c*_*L*_ mRNA copies of SARS-CoV to the respiratory tract and mucous membranes, respectively.

In summary, the ranges of three parameters were investigated in the study, namely the largest virus-containing droplet size *d*_*g*_ (four values; 50, 100, 150 and 200 μm); products of the viral load coefficient and dose–response parameters in respiratory tracts *η*_*r*_*c*_*L*_ (26 values, 10^−1^–10^4^/mRNA copy) and on mucous membranes *η*_*m*_*c*_*L*_ (26 values, 10^−4^–10^1^/mRNA copy). As *η*_*r*_ and *η*_*m*_ were assumed to be 10^−1^–10^1^ and 10^−4^–10^−2^, respectively, the ratio of *η*_*r*_ to *η*_*m*_ was in the range of 10^1^–10^5^, and thus the ratio of *η*_*r*_*c*_*L*_ to *η*_*m*_*c*_*L*_ should have been in the range of 10^1^–10^5^. With several unqualified scenarios excluded, 1,744 scenarios were considered in the study. For efficient computations and accurate predictions, we ran simulations 1,000 times for each scenario.

## Results and discussions

### Spatial distributions of infection risks

Fig 3B, 3D, 3F and 3H) shows average infection risk distributions of 1,000 simulations at the end of the exposure period via the long-range airborne route and fomite route (Patterns 1, 3 and 5), respectively. Correspondingly, Figure D (iv, vi and viii) in S1 File shows those of the fomite route (Patterns 2, 4 and 6). For fair comparison, the parameters were set to be the same for the aforesaid distributions (Fig 3B, 3D, 3F and 3H and Figure D (iv, vi and viii) in S1 File).

For the long-range airborne route, the spatial distribution of infection risk (Fig 3B) was similar to that of the reported attack rates (Fig 3A), i.e., highest in the source cubicle, lower in the adjacent cubicle and lowest in the remote cubicles. Virus-containing airborne droplets were generated by the index patient in the source cubicle, leading to the highest virus concentration in the air in the source cubicle (Figure C in S1 File) and thus the highest infection risk (Fig 3B). Due to the small temperature differences between zones, two-way airflow occurred at each inner opening in the ward [35], so some virus-containing airborne droplets spread to other cubicles by airflow. As the remote cubicles were farther away from the source than the adjacent cubicle was, the airborne droplet concentrations in the former were further diluted than that in the latter (Figure C in S1 File), leading to lower infection risk (Fig 3B). With the high mechanical ventilation rates in Ward 8A, the results from both CFD simulations [20] and multi-zone modelling methods ([35] and Figure C in S1 File) show that the aerosol concentration in the source cubicle was much higher than that in the adjacent cubicle. Therefore, the difference between infection risks (Fig 3B) in the source and adjacent cubicles was very large (1:0.43 in this scenario), which was inconsistent with the small difference (1:0.80) in the reported attack rate distribution (Fig 3A). Although several studies showed the very probable evidences for the airborne transmission of SARS such as in the Amoy Gardens outbreak [19], the inconsistence suggests that the outbreak might not merely be induced by the long-range airborne route.

For the fomite route, on the whole, the infection risk distributions were influenced by HCWs’ hands and common environmental surfaces, which were important mediums to transfer viruses from the index patient to other inpatients. As HCWs’ hands usually contact patients in a certain sequence, viruses received by normal inpatients vary with their positions in the ward. In Fig 3D, 3F and 3H and Figure D(iv, vi and viii) in S1 File, the infection risk always reaches its highest value in inpatients visited by HCWs after the index patient, and then decreases in the direction of the HCWs’ routine rounds. However, inpatients had the same opportunities to contact common surfaces, such as common toilets in Ward 8A, so common surfaces reduced the difference between viruses received by each inpatient from HCWs and contributed to a uniform infection risk distribution. Except for a few visited by HCWs after the index patient, the inpatients share a similar infection risk of 0.07.

For the six routine round patterns considered here, infection risk distributions vary. In Patterns 1 and 2 (Fig 3D and Figure D(iv) in S1 File), as more HCWs examined each patient in a routine round, the transmission of viruses was enhanced and the infection risks were generally higher than those in other patterns (Fig 3F and 3H and Figure D(vi and viii) in S1 File). In Patterns 3 and 4 (Fig 3F and Figure D(vi) in S1 File), as different groups of HCWs were responsible for different cubicles, HCWs did not transmit viruses across cubicles, and thus the infection risks for inpatients in the adjacent cubicle and remote cubicles were nearly the same. In Patterns 5 and 6 (Fig 3H and Figure D(viii) in S1 File), the inpatient subsequently visited by HCWs after the index patient was not necessarily Inpatient 10 or 12. Therefore, although the infection risks still decreased in the direction of routine rounds, the reduction was small compared with other patterns (Fig 3D and 3F and Figure D(iv and vi) in S1 File).

Among the six infection risk distributions, only those of Patterns 1 and 5 (Fig 3D and 3H) were highest in the source cubicle, lower in the adjacent cubicle, and lowest in the remote cubicles, similar to that of the reported attack rates (Fig 3A). Nevertheless, as for Pattern 1, the difference between infection risks (Fig 3D) in the source and adjacent cubicles was too large (1:0.35 in this scenario) and not consistent with the small difference (1:0.80) in the reported attack rate distribution (Fig 3A). In contrast, for Pattern 5, the difference between infection risks (Fig 3H) in the source and remote cubicles was too small (1:0.52 in this scenario), not consistent with the large difference (1:0.28) in the reported attack rate distribution (Fig 3A).

### The hypotheses with the best fitness in different scenarios

**Fig 4:**
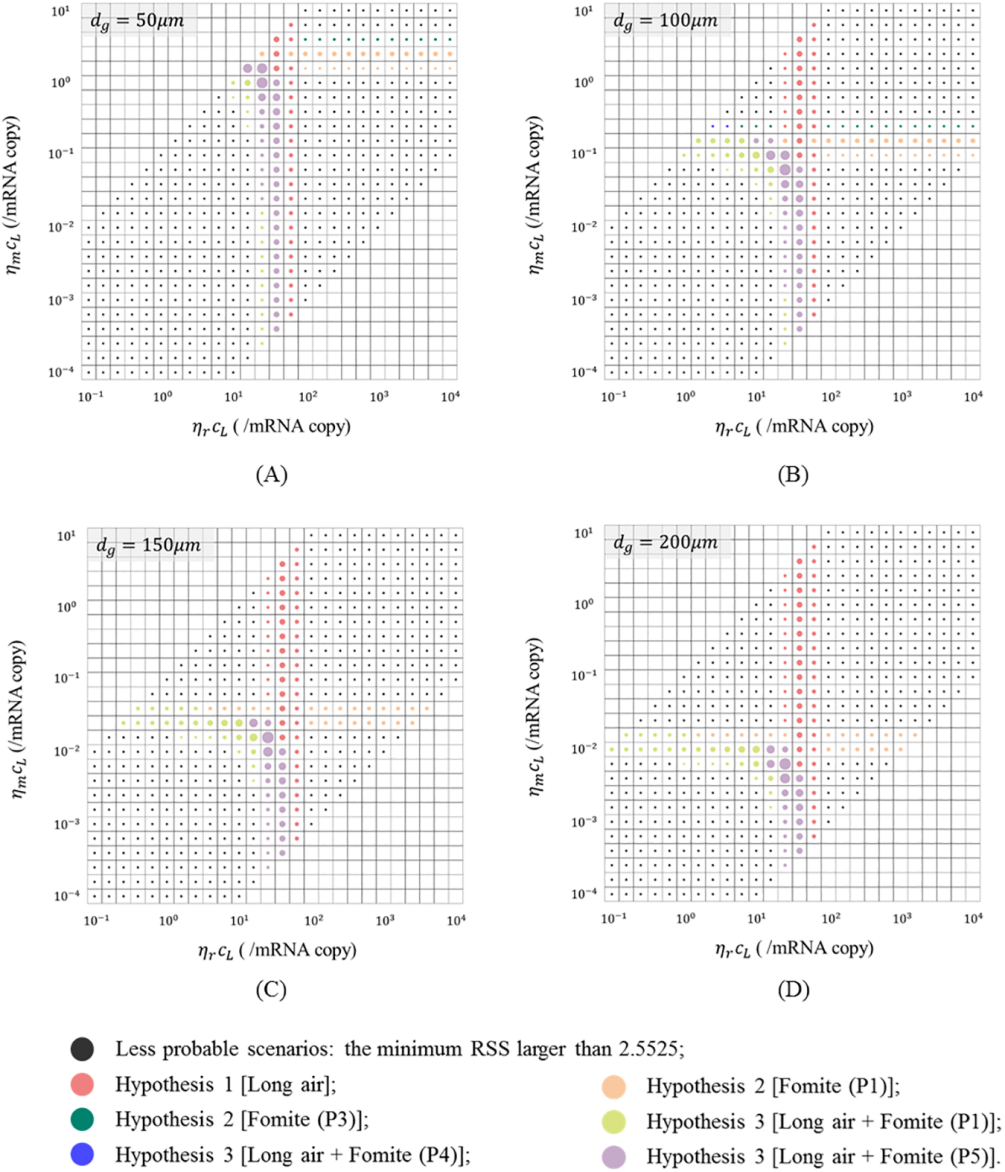
Illustration of the hypotheses with the minimum RSS in 1,744 scenarios. *d*_*g*_ denotes the largest virus-containing droplet size (4 values: 50, 100, 150 and 200 μm); *η*_*r*_*c*_*L*_ and *η*_*m*_*c*_*L*_ denote products of viral load coefficient and dose–response parameters in respiratory tracts (26 values, 10^−1^–10^4^/mRNA copy) and on mucous membranes (26 values, 10^−4^–10^1^/mRNA copy). (A) *d*_*g*_ = 50 μm; (B) *d*_*g*_ = 100 μm; (C) *d*_*g*_ = 150 μm; (D) *d*_*g*_ = 200 μm. Dots of different colours represent different hypotheses as shown in the legend. Dot diameter is inversely proportion to the value of RSS. The smallest RSS in all scenarios (the biggest dot) was 0.5505, and the small black dots represent scenarios with the minimum RSS at least five times as much as 0.5505.

Fig 4 shows the hypotheses with the best fitness (the minimum RSS) in the 1,744 scenarios. Fig 4 shows that six kinds of non-black dots, representing Hypotheses 1 [Long air] (red dots), 2 [Fomite (P1)] (orange dots), 2 [Fomite (P3)] (green dots), 3 [Long air + Fomite (P1)] (cyan dots), 3 [Long air + Fomite (P4)] (blue dots) and 3 [Long air + Fomite (P5)] (purple dots), respectively. Since the distribution of reported attack rates in this SARS outbreak exhibited a statistically significant pattern (*P* = 0.0015, Pearson chi-square test), in many scenarios the minimum RSS were larger than 2.5525 (small black dots in Fig 4), indicating large deviations from the outbreak data. Therefore, these scenarios were regarded as less probable ones.

In Fig 4, when the product of dose–response parameters in mucous membranes and viral load coefficient *η*_*m*_*c*_*L*_ was very large, the prediction of Hypothesis 1 [Long air] (red dots) fitted best with the reported data. As the exposure for this hypothesis occurred only in the respiratory tract, the value of RSS did not vary with dose–response parameters on mucous membranes *η*_*m*_. Thus, large *η*_*m*_ values would have led to overly high infection risks under Hypotheses 2 and 3, but would not have influenced the long-range airborne route. Similarly, when the product of dose–response parameters in respiratory tracts and viral load coefficient *η*_*r*_*c*_*L*_ was very large, the prediction of Hypothesis 2 [Fomite] (orange and green dots) fitted best with the reported data. When *η*_*r*_*c*_*L*_ and *η*_*m*_*c*_*L*_ were relatively small, the prediction of the Hypothesis 3 [Long air + Fomite] (cyan, blue and purple dots) fitted best with the reported data.

In Fig 4, the viral load coefficient is larger than 1 in over 95% of more probable scenarios (non-black dots), meaning that the viral load for the index patient in the computation was very probable to be higher than the measured data of ordinary SARS patients [33]. Assuming that the dose–response parameter on mucous membranes *η*_*m*_ was 3.2 × 10^−3^/mRNA copy [40] and that parameter in respiratory tracts *η*_*r*_ was 10^3^ times higher than that on the mucous membranes, i.e., 3.2/mRNA copy [41], all of the viral load coefficients for more probable scenarios (non-black dots) were larger than 1.97. The high viral load coefficients support other studies suggesting that the index patient was a super-spreader [42–44].

As shown in Tables 1 and 2, the minimum RSS of other patterns in Hypotheses 3 [Long air + Fomite (P1, P2, P3, P4, P5 and P6)], 0.7092, 0.9762, 0.7790, 0.7514, 0.5105 and 0.7675, are smaller than those of the two single-route modes, 1.0394 at least. Thus, Hypothesis 3 [Long air + Fomite] was more possible than the two single-route hypotheses. Moreover, among the 6 patterns in Hypothesis 3, the minimum RSS of Pattern 5 was smallest (0.5105), indicating that it was the most possible.

**Table 1:** Important parameters for scenarios with the minimum RSS for Hypotheses 1 [Long air] and 2 [Fomite (including six patterns)].

Parameter		Reported data	Hypothesis 1 [Long air]	Hypothesis 2 [Fomite (including six patterns)]					
				F(P1)	F(P2)	F(P3)	F(P4)	F(P5)	F(P6)
**The minimum RSS**		N.A.	1.0394	1.2638	2.5947	1.8139	2.0042	2.3405	3.3971
***d*_*g*_^1^ (μm)**		Unknown	-	100	100	150	150	150	200
***η***_***r***_***c***_***L***_^2^ **(/mRNA copy)**		Unknown	10^1.6^	-	-	-	-	-	-
***η***_***m***_***c***_***L***_^2^ **(/mRNA copy)**		Unknown	-	10^−0.8^	10^−0.8^	10^−1.4^	10^−1.4^	10^−1.4^	10^−1.8^
**Average infection risk**	Source ward	0.6500	0.7942	0.5842	0.5159	0.5635	0.5228	0.4535	0.4181
	Adjacent ward	0.5238	0.3701	0.3816	0.3061	0.3628	0.3563	0.4275	0.3697
	Remote wards	0.1818	0.1197	0.3170	0.3669	0.3548	0.3529	0.3682	0.4033
	Overall	0.4054	0.3731	0.4076	0.3899	0.4135	0.3998	0.4081	0.3978
**Relative contribution**	Long-range airborne	Unknown	100%	0	0	0	0	0	0
	Fomite	Unknown	0	100%	100%	100%	100%	100%	100%

1. *d*_*g*_ denotes the largest virus-containing droplet size

2. *η*_*r*_*c*_*L*_ and *η*_*m*_*c*_*L*_ denote products of viral load coefficient and dose–response parameters in respiratory tracts and on mucous membranes, respectively.

**Table 2:** Important parameters for scenarios with the minimum RSS for Hypothesis 3 [Long air + Fomite (including six patterns)].

Parameter		Reported data	Hypothesis 3 [Long air + Fomite (including six patterns)]					
			L+F(P1)	L+F(P2)	L+F(P3)	L+F(P4)	L+F(P5)	L+F(P6)
**The minimum RSS**		N.A.	0.7092	0.9762	0.7790	0.7514	0.5105	0.7675
***d*_*g*_^1^ (μm)**		Unknown	50	200	100	150	100	100
***η***_***r***_***c***_***L***_^2^ **(/mRNA copy)**		Unknown	10^1.2^	10^1.4^	10^1.4^	10^1.4^	10^1.4^	10^1.4^
***η***_***m***_***c***_***L***_^2^ **(/mRNA copy)**		Unknown	10^0.2^	10^−2.4^	10^−1.2^	10^−1.8^	10^−1.2^	10^−1.2^
**Average infection risk**	Source ward	0.6500	0.7181	0.7578	0.7599	0.7503	0.7286	0.7019
	Adjacent ward	0.5238	0.3799	0.3488	0.3781	0.3754	0.4172	0.3560
	Remote wards	0.1818	0.2426	0.2307	0.2277	0.2268	0.2357	0.2314
	Overall	0.4054	0.4101	0.4067	0.4142	0.4105	0.4204	0.3939
**Relative contribution**	Long-range airborne	Unknown	40%	58%	56%	57%	57%	63%
	Fomite	Unknown	60%	42%	44%	43%	43%	37%

1. *d*_*g*_ denotes the largest virus-containing droplet size

2. *η*_*r*_*c*_*L*_ and *η*_*m*_*c*_*L*_ denote products of viral load coefficient and dose–response parameters in respiratory tracts and on mucous membranes, respectively.

In Table 2, in scenarios with the minimum RSS for Hypothesis 3 [Long air + Fomite], the fomite route plays a non-negligible role in transmission, contributing at least 37% to the infection risk. Except for Pattern 1, the long-range airborne route was predominant, which is consistent with several findings of the similarity between bio-aerosol concentrations and reported attack rates distributions in SARS outbreaks [19–21].

### Limitations

This study had three main limitations. First, most of the human behaviour was assumed in the multi-agent model (Tables G and H in S1 File) because relevant descriptions were not available in the literature. The information of human behaviours were very important for our model. As shown in this study, the routine round patterns of doctors and nurses influenced the infection risk patterns (Fig 3 and Figure D in S1 File) and hypothesis probabilities (Fig 4, Tables 1 and 2). In addition, the diversity of modes of HCWs visiting patients was not considered in this study. Different patients might have required different frequencies, intensities, or HCWS visiting patterns, which might bring in more deviations in infection risk distributions. In future, more detailed information about human behaviours of representative people such as patients, HCWs and visitors in healthcare settings should be reported for outbreaks of infectious diseases.

Second, some parameters for the biological properties of SARS-CoV in the multi-agent model were not available, such as the transfer rates between surfaces (Table C in S1 File) and the first-order inactivation rates in the air and on surfaces (Table D in S1 File), and were estimated or replaced by those of other viruses or even bacteria. Moreover, several parameters such as dose response parameters might be variable from patient to patient, but the individual differences were not considered in this study, which decreases the diversity of predicted infection risk distributions. Experimental investigations of SARS-CoV are lacking mainly because of safety considerations [18]; and several authors have suggested 229E, a low-virulence human coronavirus [45], as a surrogate [18, 40]. In future, more experimental measurements of parameters for the biological properties of SARS-CoV or the surrogate, 229E, are needed.

Third, due to the lack of information, the length of exposure period were assumed to be the same for all susceptible patients, and the index patient was assumed to be the only source. However, different patients might have different timings of admission, discharge, or symptom onset, and thus different exposure periods. Ignoring the individual differences in the exposure periods reduces the diversity of infection risk distributions, and leads to omission of several possible scenarios. Moreover, the transmission from early-onset cases to the later cases might have occurred, according to the illness onset dates reported by Li et al. [20]. Neglecting the exposure doses caused by these early-onset cases results in underestimation of infection risk for other cases, which affects the distribution patterns of infection risk in the ward. In future, more detailed information about the timing for patients should be recorded in outbreak reports of infectious diseases.

## Conclusions

In this study, a mechanism-based investigation was conducted to explore the role of the fomite route in the transmission of SARS-CoV infection. The results could help to recommend appropriate infection control measures in a focused manner. Based on the simulation results and analyses, the following conclusions can be drawn under our assumed conditions.

- In our investigated scenarios, for most of the routine round patterns, SARS-CoV was less probable to transmit via the fomite route alone. The virus might have spread via the long-range airborne route alone, but it was more probable that the virus could transmit in combined routes, especially when the viral loads and dose–response parameters were relatively small.
- It’s found that the index patient was very probable to generate more viruses than ordinary SARS patients, which supported the perception that the patient was a super-spreader.
- In the very probable combined routes, the fomite route played a non-negligible role. For most patterns, the airborne route was predominant.
- Doctors and nurses were found to be the most possible to conduct their routine rounds following Pattern 5 (examining inpatients randomly in the clockwise direction).

## Supporting information

S1 File The following information is described in detail, e.g. details of the mathematical models, parameter selections for the mathematical models and supplemental figures. (DOCX)
