# Supplementary material for: Role of fomites in SARS transmission during the largest hospital outbreak in Hong Kong
Source: PLoS One. 2017 Jul 20;12(7):e0181558. doi: 10.1371/journal.pone.0181558 (PMC5519164; doi:10.1371/journal.pone.0181558)
Supplement: S1 File — (DOCX) [file pone.0181558.s001.docx]

**Supplementary information**

**Possible evidence for the fomite transmission in the largest SARS hospital outbreak in Hong Kong**

Shenglan Xiao^1*^, Yuguo Li^1^, Tze-wai Wong^2^, David SC Hui^3^

^1^Department of Mechanical Engineering, The University of Hong Kong, Pokfulam Road, Hong Kong SAR, China

^2^JC School of Public Health and Primary Care, The Chinese University of Hong Kong, Prince of Wales Hospital, Shatin, Hong Kong SAR, China

^3^Department of Medicine and Therapeutics, The Chinese University of Hong Kong, Prince of Wales Hospital, Shatin, Hong Kong SAR, China

**SI A. Details of the mathematical models**

**A1 The multi-zone model**

The multi-zone model [1–3] was applied to calculate aerosol concentration distributions in Ward 8A. As suggested by Chen et al. [3], the ward was divided into six zones, with every cubicle set as an individual zone and the long corridor divided into two zones (Figure A). The volumes, mechanical ventilation rates and heat gains for the six zones are summarized in Table A.


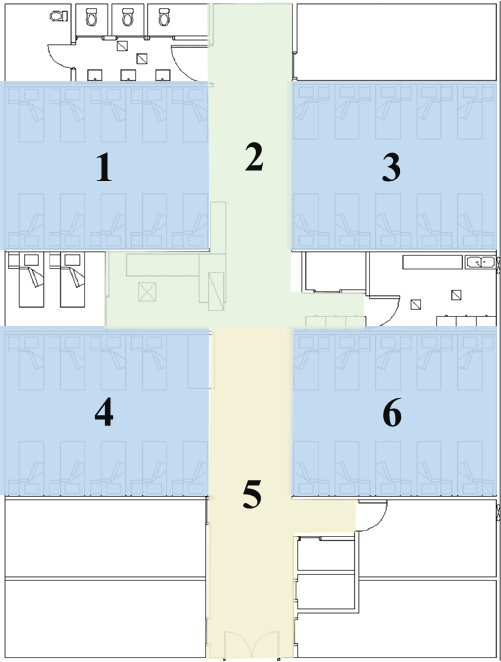


**Figure A.** The division of Ward 8A into six zones for multi-zone modelling. The long corridor was divided into two zones.

The multi-zone model was based on three macroscopic conservation equations: airflow balance, energy balance and aerosol mass balance [1, 3]. Regardless of wind effect, the airflow patterns in the ward were influenced by mechanical ventilation and thermal buoyancy. The index patient was assumed to be a steady source. The aerosols were modelled as passive tracers that did not affect the flow [4].

Figure B shows the procedures in the multi-zone model program. First, the initial temperatures in each zone were set and the model MIX2 (Multi-zone Infiltration and eXfiltration) [1] was used to solve nonlinear equations based on the airflow balance and acquire the airflow rates. According to the calculated airflow rates, new temperatures were then obtained by solving the energy equations and compared with the old temperatures. If the temperatures had not reached convergence, the initial temperature was updated and the iterations continued; if the temperatures converged to a set of constants, the airflow pattern was calculated by MIX2. Finally, the aerosol concentrations were calculated based on the aerosol balance.


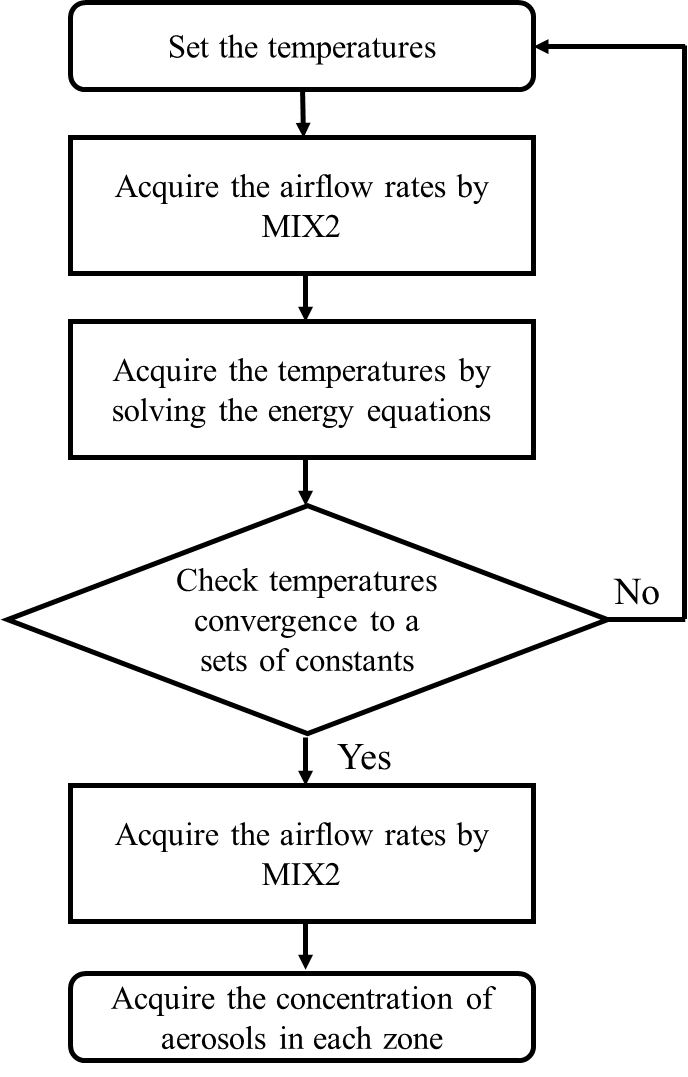


**Figure B.** The major procedures of the multi-zone model.

**A2 The long-range airborne route exposure model**

Following Gao [5], the exposure dose for the susceptible individuals in Zone *j* during the exposure time of *T* due to the airborne route in the respiratory tract was denoted as$D_{a}(T,j)$ and was estimated as

| $D_{a}(T,j)=\int_{0}^{T} \int_{0}^{d_{a}} C_{a}\left( t,j \right)p \delta\left( d \right)\frac{1}{6}\pi d_{0}^{3}L_{a}\left( d,j \right)f_{a}\left( d,j \right)d(d)dt$ | (S1) |
| --- | --- |

where $d_{0}$ is the droplet’s initial diameter, $d$ is the final diameter after complete evaporation, and $d_{a}$ is the largest final diameter for airborne droplets; $C_{a}\left( t,j \right)$ is the number concentration of all the airborne droplets in the air of Zone *j* at time point *t*; $p$ is the pulmonary ventilation rate; $\delta\left( d \right)$ is the deposition rate of droplets of diameter $d$ in the respiratory tract from the deposition model of ICRP [6]; $L_{a}\left( d,j \right)$ is the concentration of viable viruses (TCID50/ml or genome copies/ml) with the final diameter of $d$ in inhaled droplets of the susceptible individuals in Zone *j*; and $f_{a}\left( d,j \right)$ is the probability distribution function of the final droplet size in the inhaled air of the susceptible individuals in Zone *j*.

According to Xie et al. [7] and Liu et al. [8], the evaporation time for airborne droplets is less than 0.1 second and their size decreases by one third on evaporation. Therefore, we assumed that in all zones, the inhaled airborne droplets have evaporated, and the diameters would be one third of their initial diameter, namely ${d=d}_{0}/3$. After evaporation, virus viability in the droplets falls sharply to one quarter of its initial value and then declines slowly [9], so we assumed that the final concentration of viable viruses in airborne droplets was one quarter the initial concentration, namely $L_{a}\left( d,j \right)=\frac{1}{4}L$, where $L_{0}$ is the initial concentration of viable viruses (TCID50/ml or genome copies/ml) and is assumed to be irrelevant to the initial diameter of $d_{0}$ in expired droplets.

The index patient was assumed to cough with a frequency ($f_{c}$) of 12 times an hour [10], and generate $N_{c}$ (= 2000) droplets with each cough [11], so the droplet generation rate was $f_{c}N_{c}$. According to the probability distribution function of the initial expired droplets $f\left( d_{0} \right)$ given by Atkinson and Wein [12], a droplet generation rate for diameters between $d_{01}$ and $d_{02}$ can be expressed as $f_{c}N_{c}\int_{d_{01}}^{d_{02}} f\left( d_{0} \right)d(d_{0})$. Using the deposition loss rate coefficients from Thatcher et al. [13], the compositions of inhaled airborne droplets can be acquired by the multi-zone methods, so $C_{a}\left( t,j \right)f_{a}\left( d,j \right)$ in Equation S1 could be calculated. Therefore, Equation S1 could be simplified as

| $D_{a}(T,j)=\frac{9}{8}\pi pL_{0}\int_{0}^{T} \int_{0}^{d_{a}} \delta\left( d \right)d^{3}f_{a}\left( d,j \right)C_{a}\left( t,j \right)d(d)dt$ | (S2) |
| --- | --- |

**A3 The surface contamination model**

The fomite transmission of disease is thought to be induced by touching contaminated surfaces, including the hands of the index patient [14]. In this study, surface contamination was initialised by the deposition of expired droplets from the index patient. In this study, surfaces around the index patient were assumed to be steady virus sources. The behaviour frequencies and surface touching sequences listed in Tables G and H were assumed or estimated from life experience and observation studies.

Regardless of virus concentration diversity, we assumed that the virus concentration would stay uniform on the same surface. According to Plipat et al. [15], virus quantities on the hand $N_{h}^{'}$ and the environmental surface $N_{s}^{'}$ after a touching action can be calculated as

| $N_{h}^{'}=\alpha_{sh}\cdot\frac{N_{s}}{A_{s}}\cdot A_{c}-\alpha_{hs}\cdot\frac{N_{h}}{A_{h}}\cdot A_{c}+N_{h}$ = $\frac{A_{c}}{A_{s}}\cdot\alpha_{sh}\cdot N_{s}+(1-\frac{A_{c}}{A_{h}}\cdot\alpha_{hs}) \cdot N_{h}$ | (S3) |
| --- | --- |
| $N_{s}^{'}=\alpha_{hs}\cdot\frac{N_{h}}{A_{h}}\cdot A_{c}-\alpha_{sh}\cdot\frac{N_{s}}{A_{s}}\cdot A_{c}+N_{s}$ = $\frac{A_{c}}{A_{h}}\cdot\alpha_{hs}\cdot N_{h}+(1-\frac{A_{c}}{A_{s}}\cdot\alpha_{sh}) \cdot N_{s}$ | (S4) |

where $\alpha_{sh}$ and $\alpha_{hs}$ are respectively the transfer rates from the environmental surface to the hand, and from the hand to the environmental surface; $N_{s}$ and $N_{h}$ are respectively the virus quantities on the environmental surface and the hand before touching; $A_{s}$ and $A_{h}$ are respectively the area of the environmental surface and the hand; and $A_{c}$ is the contact area.

Equation S3 and S4 can be written in matrix form as

| $\left[ \begin{matrix} N_{h}^{'} & N_{s}^{'} \end{matrix} \right]=\left[ \begin{matrix} N_{h} & N_{s} \end{matrix} \right]\left[ \begin{matrix} 1-\frac{A_{c}}{A_{h}}\alpha_{hs} & \frac{A_{c}}{A_{h}}\alpha_{hs} \\ \frac{A_{c}}{A_{s}}\alpha_{sh} & 1-{\frac{A_{c}}{A_{s}}\alpha}_{sh} \end{matrix} \right]$ | (S5) |
| --- | --- |

As shown in Equations S3 and S4, virus quantities on surfaces after one touching action only depend on the state before the action rather than the sequence of states that preceded it, which conforms to the definition of the Markov chain [16]. Therefore, every behaviour consisting of a series of touching actions can be regarded as a discrete-time Markov chain, and surfaces (including environmental surfaces and hands) can be regarded as different states in the Markov chain. The square matrix in Equation S5 can be regarded as a simple transition matrix for the Markov chain.

On the outbreak floor, we assumed there are *l* representative surfaces (including environmental surfaces and hands). For one type of behaviour including *n* touching actions, taking the virus inactivation on surfaces into consideration, we could obtain the final conditions:

| ${\underline{\underline{N}}}^{(n)}={\underline{\underline{N}}}^{\left( n-1 \right)}{\underline{\underline{P}}}^{(n)}\underline{\underline{E}}={\underline{\underline{N}}}^{\left( 0 \right)}{\underline{\underline{P}}}^{(1)}{\underline{\underline{P}}}^{(2)}\cdots{\underline{\underline{P}}}^{(n)}\underline{\underline{E}}={\underline{\underline{N}}}^{\left( 0 \right)}\prod_{k=1}^{n} {\underline{\underline{P}}}^{(k)}\underline{\underline{E}}$ | (S6) |
| --- | --- |
| with ${\underline{\underline{N}}}^{(k)}=[N_{1}^{\left( k \right)},\cdots,N_{l}^{(k)}]$, *k=0, ..., n,*   \| 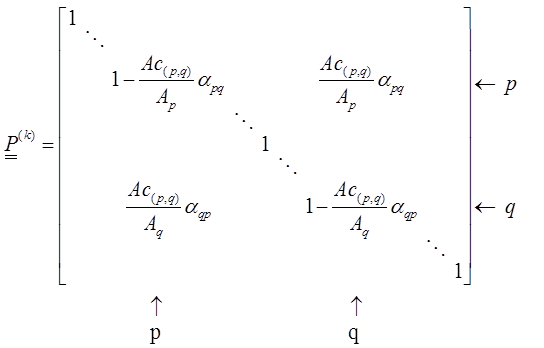, *k=1, ...,n*.  and $\underline{\underline{E}}= diag\left( e^{-b_{1}\cdot\Delta T}, e^{-b_{2}\cdot\Delta T}, \ldots, e^{-b_{l}\cdot\Delta T} \right).$ \| \| --- \| |  |

where ${\underline{\underline{N}}}^{(k)}$ indicates a row vector of $l$ elements, representing the virus quantities on surfaces after the *k*th contact;$N_{x}^{\left( k \right)}$ refers to virus quantities on the *x*th surface after the *k*th contact; ${\underline{\underline{P}}}^{(k)}$ is the transition matrix with a dimension of $l\times l$ in the *k*th contact, when the *p*th surface contacts the *q*th surface; $\alpha_{xy}$ is the transfer efficiency from the *x*th surface to the *y*th surface; $A_{x}$ is the area of the *x*th environmental surface, and ${Ac}_{(x,y)}$ is the contact area when the *x*th surface contacts with the *y*th surface; $\underline{\underline{E}}$ is a diagonal matrix with the dimension of $l\times l$ to indicate the effect of virus inactivation on surfaces; $b_{x}$ is the virus inactivation rate on the *x*th surface; and$\Delta T$ is the time duration between the prior behaviour and the present one.

The surfaces of the index patient and mucous membranes are special surfaces so there were some special treatments for them. As virus concentrations on surfaces of the index patient were assumed to be steady, if in the *k*th touching action, the *p*th surface pertained to the index patient, then the value of $b_{p}$ in $\underline{\underline{E}}$ was 0, and the value of $\frac{{Ac}_{(p,q)}}{A_{p}}\alpha_{pq}$ and $\frac{{Ac}_{(p,q)}}{A_{q}}\alpha_{qp}$ in the *p*th column of transition matrix ${\underline{\underline{P}}}^{(k)}$ was 0. On the other hand, in this study, it was assumed that no virus would transfer from mucous membranes to hands and viruses on mucous membranes would not naturally die, so if the *q*th surface is a mucous membrane, the values of $b_{q}$ in $\underline{\underline{E}}$ would be 0, and the values of $\frac{{Ac}_{(p,q)}}{A_{q}}\alpha_{qp}$ in the *q*th row of the transition matrix ${\underline{\underline{P}}}^{(k)}$ would be 0.

The exposure dose for Individual *i* during exposure time *T* due to the fomite route was denoted as$D_{f}(T,i)$ and could be estimated as

| $D_{f}\left( T,i \right)=N_{m}(T,i)$ | (S7) |
| --- | --- |

where $N_{m}(T,i)$ indicates the virus quantities on the mucous membranes of Individual *i* after exposure time *T*.

**A4 The dose–response relationship model**

According to Gao [5], the infection risk of an individual in the *i*th hypothesis ${IR}_{i}$ (*i* = 1, 2 and 3) can be calculated as

| ${IR}_{1}=1-e^{-\eta_{r}D_{a}}$ | (S8-1) |
| --- | --- |
| ${IR}_{2}=1-e^{-{\eta_{m}D}_{f}}$ | (S8-2) |
| ${IR}_{3}=1-e^{-\eta_{r}D_{a}-{\eta_{m}D}_{f}}$ | (S8-3) |

where $\eta_{r}$ and $\eta_{m}$ are dose–response parameters in the respiratory tract and on mucous membranes, respectively.

**SI B. Parameter selection**

**Table A.** Parameters for the multi-zone model.

| **Parameter** | **Zone 1** | **Zone 2** | **Zone 3** | **Zone 4** | **Zone 5** | **Zone 6** | **Data source** |
| --- | --- | --- | --- | --- | --- | --- | --- |
| **Volume (m^3^)** | 121.5 | 97.2 | 121.5 | 121.5 | 97.2 | 121.5 | Estimated [4] |
| **Supply airflow rate (m^3^/s)** | 0.336 | 0.049 | 0.290 | 0.305 | 0 | 0.310 | [4] |
| **Exhaust airflow rate (m^3^/s)** | 0 | 0.714 | 0 | 0 | 0.576 | 0 | [4] |
| **Heat gain (kW)** | 2.310 | 1.239 | 2.310 | 2.234 | 1.239 | 2.478 | Estimated [3] |

**Table B.** The material types and areas of surfaces.

| **Surface** | **Material type** | **Area*^a^* (cm^2^)** | **Data source** |
| --- | --- | --- | --- |
| Clothes | Porous surfaces | 10,000 | Estimated [17] |
| Bed surface |  | 18,000 | Assumed |
| Curtains |  | 1,000 | Assumed |
| Over-bed table | Non-porous surfaces | 4,800 | Assumed |
| Bedside table |  | 8,400 | Assumed |
| Cup |  | 250 | Assumed |
| Bed rail |  | 2,355 | Assumed |
| Water heater button |  | 3 | Assumed |
| Toilet door handle (outer) | Toilet surfaces | 47 | Assumed |
| Toilet door handle (inner) |  | 47 | Assumed |
| Toilet lid |  | 500 | Assumed |
| Toilet flush buttons |  | 3 | Assumed |
| Toilet taps |  | 3 | Assumed |
| Toilet sanitizer button |  | 3 | Assumed |
| Hand contact area | Skin | 40 | Estimated [18, 19] |
| Finger contact area |  | 2 | Estimated [20] |
| Non-mucosal regions of head and neck |  | 1,300 | Estimated [17] |
| Mucous membranes | Mucous membranes | 10 | Assumed [5] |

**a** Surface areas are effect areas that are commonly touched rather than the actual areas.

**Table C.** Transfer rates between surfaces of different materials.

| **Donor surface** | **Acceptor surface** | **Transfer rate** | **Data source*^a^*** |
| --- | --- | --- | --- |
| Hand | Porous surface | 17% | *Staphy. saprophyticus*, from hand to fabric [21, 22] |
| Hand | Skin | 17% | Rhinovirus, from finger to finger [22] |
| Hand | Non-porous surface | 14% | Rhinovirus, from hand to brass door knob [22] |
| Hand | Toilet surface | 36% | Rhinovirus, from hand to faucet handle [22] |
| Hand | Mucous membranes | 34% | PRD-1, from hand to mouth [23]  PRD-1 (6), from hand to lips/skin [22] |
| Porous surface | Hand | 0.3% | MS-2, from cotton to hand [24] |
| Skin | Hand | 17% | Rhinovirus, from finger to finger [22] |
| Non-porous surface | Hand | 37% | MS-2, from stainless steel to hand [24] |
| Toilet surface | Hand | 16% | Rhinovirus, from faucet handle to hand [22] |
| Mucous membranes | Hand | 0% | Assumed |

**a** The transfer rates of SARS-CoV between hands and surfaces were not available in the literature, so the data for other viruses and bacteria were used as surrogates. Data from the same researcher [22, 24] were selected to maintain consistency.

**Table D.** First-order inactivation rates at different sites.

| **Site** | **Value** | **Data source** |
| --- | --- | --- |
| In air | 0.03/hr | Estimated, 229E coronavirus***^a^***, in aerosols, 20°C, 30% RH [25] |
| On porous surface | 0.04/hr | Assumed***^b^*** |
| On skin | 0.80/hr | Estimated, 229E coronavirus***^a^***, on hands [26] |
| On non-porous surface | 0.04/hr | Estimated, SARS-CoV, on plastic surfaces, dried [27] |
| On toilet surface | 0.46/hr | Estimated, SARS-CoV, on plastic surfaces, 80–85% RH [28] |

**a** The first-order inactivation rate of SARS-CoV on skin was not available in the literature; 229E coronavirus was used as a surrogate.

**b** The first-order inactivation rate of SARS-CoV on porous surfaces was not available in the literature and was assumed to be equal to that on non-porous surfaces following Duan et al. [29].

**Table E.** Virus sources at different sites

| **Parameter** | **Sites *^a^*** | **Value** | **Data source** |
| --- | --- | --- | --- |
| $L_{0}$ | In initially expired droplets | 10^3^–10^9^ mRNA copies/ml***^b^*** | Viral loads in nasopharyngeal aspirates [30] |
| $L_{s}$ | On skin | 2.6×10^-8^$L_{0}$ mRNA copies/cm^2^ | Estimated***^c^*** |
| $L_{p}$ | On porous surfaces | 3.7×10^-7^$L_{0}$ mRNA copies/cm^2^ | Estimated***^c^*** |
| $L_{np}$ | On non-porous surfaces | 3.7×10^-7^$L_{0}$ mRNA copies/cm^2^ | Estimated***^c^*** |

**a** These sites all refer to those associated with the index patient.

**b** The index patient developed a fever on February 24, 2003 [31], so the computational period (March 4–12, 2003) comprised days 9–17 after onset of symptoms. According to Peiris et al. [30], the viral loads ranged from 10^3^ to 10^9^ mRNA copies/ml.

**c** It is assumed that when the index patient coughed, airborne droplets suspended in the air and other droplets were uniformly deposited on a small area around him. The virus concentrations on different surfaces vary with the first-order inactivation rates of the surface materials and the largest diameter for virus-containing droplets $d_{g}$. In Table S5, $d_{g}$ is set to be the baseline value, 100 μm.

**Table F.** Dose–response parameters at different exposure sites

| **Parameter** | **Exposure site** | **Value** | **Data source** |
| --- | --- | --- | --- |
| $\eta_{m}$ | Mucous membranes | 3.2×10^-3^/PFU***^a^*** | Estimated [32] |
| $\eta_{r}$ | Respiratory tract | 3.2/PFU | Assumed***^b^*** |

**a** The unit of dose–response parameters of SARS-CoV is not consistent with that of source strength due to a lack of related data. A PFU/RNA ratio cannot be determined because the ratio would vary with virus species and environmental condition in the experiments.

**b** The dose–response parameter of SARS-CoV on the respiratory tract was not available in the literature, and was assumed to be 10^3^ times higher than that on the mucous membranes, similar to the A2 influenza virus [12].

**Table G.** Behaviour frequencies

| **Behaviour** | **Executors** | **Frequency** | **Data source** |
| --- | --- | --- | --- |
| Touching one’s own clothes | All agents | 3/hr | Assumed |
| Touching one’s own non-mucosal regions of head and neck | All agents | 13/hr | Estimated [33] |
| Touching one’s own mucous membranes | Nurses and the health assistant | 9/hr | Estimated [34] |
|  | Doctors and medical students | 5/hr | Estimated [34] |
|  | Patients, visitors and cleaning staff | 16/hr | Estimated [34] |
| Touching one’s own bed rails | Patients | 10/day | Assumed |
| Touching one’s own over-bed tables | Patients | 3/day | Assumed |
| Touching one’s own bed surfaces | Patients | 3/hr | Assumed |
| Touching one’s own bedside table | Patients | 3/day | Assumed |
| Helping the index patient fetch water | The health assistant | 2.5/day | [35] |
| Helping the index patient with urination | The health assistant | 4–7/day | [36] |
| Helping the index patient with defecation | The health assistant | 1/3–3/day | [37] |
| Fetching water | Normal patients | 2.5/day | [35] |
| Urination | Normal patients | 4–7/day | [36] |
| Defecation | Normal patients | 1/3–3/day | [37] |
| Visiting patients | Visitors | 1/day | Assumed |
| Medical examinations | Doctors | Beginning at 08:00 | Assumed |
| Clinical assessment | Medical students | March 6, 2003 and March 7, 2003 | [38] |
| Routine rounds | Nurses | Beginning at 03:00, 07:00, 11:00, 15:00, 19:00 and 23:00 ***^a^*** | Assumed |
| Cleaning cubicles | Cleaners | Beginning at 08:00 and 18:00 | Assumed |
| Cleaning toilets | Cleaners | Beginning at 08:00 and 18:00 | Assumed |

**a** Healthcare workers would generally take observation for every patient (such as pulse, temperature and blood pressure) every 4 hours, namely 6 times per day.

**Table H.** Assumed sequences of touching surfaces in behaviours

| **Behaviour** | **Executors** | **Assumed sequence of touching surfaces*^a^*** |
| --- | --- | --- |
| Touching one’s own clothes | All agents | Clothes |
| Touching one’s own non-mucosal regions of head and neck | All agents | Non-mucosal regions of head and neck |
| Touching one’s own mucous membranes | All agents | Mucous membranes |
| Touching one’s own bed rails | Patients | Bed rails |
| Touching one’s own over-bed tables | Patients | Over-bed tables |
| Touching one’s own bed surfaces | Patients | Bed surfaces |
| Touching one’s own bedside table | Patients | Bedside tables |
| Helping the index patient fetch water | The health assistant | Cup → water heater button → water heater button → cup |
| Helping the index patient with urination/defecation | The health assistant | Toilet door handle (outer) → toilet door handle (inner) → toilet lid → toilet lid → toilet flush button → toilet tap → toilet sanitizer button → toilet door handle (inner) → toilet door handle (outer) |
| Fetching water | Normal patients | Bed surface → bed rails → cup → water heater button → water heater button → cup → bed rails → bed surface |
| Urination/defecation | Normal patients | Bed surface → bed rails → toilet door handle (outer) → toilet door handle (inner) → toilet lid → toilet lid → toilet flush button → toilet tap → toilet sanitizer button → toilet door handle (inner) → toilet door handle (outer) → bed rails → bed surface |
| Visiting patients/medical examinations/clinical assessment routine rounds/ | Visitors/doctors/medical students/  nurses | Curtains → bed rails → bed surfaces → clothes → patients’ hands → clothes → bed surfaces → bed rails → curtains |
| Cleaning cubicles | Cleaners | Curtains → over-bed table***^b^*** → bedside table***^b^*** → curtains |
| Cleaning toilets | Cleaners | Toilet outside door handle → toilet inside door handle → toilet lid***^b^*** → toilet lid***^b^*** → toilet flush button***^b^*** → toilet inside door handle → toilet outside door handle |

**a** HCWs’ surface touching sequences were assumed based on YouTube videos of student-focused training in healthcare from the Arizona Medical Training Institute (AZMTI) (<https://www.youtube.com/user/AZMTI>).

**b** Underlining indicates surfaces that cleaning staff would clean.

**Table I.** Other parameters

| **Parameter** | **Description** | **Value** | **Data source** |
| --- | --- | --- | --- |
| $T$ | Computational duration | 9 days, from March 4 to 12, 2003 | Assumed [31] |
| $d_{a}$ | Largest diameter for airborne droplets | 10 μm | [10, 39] |
| $d_{i}$ | Largest diameter for inspirable droplets | 100 μm | [10] |
| $d_{g}$ | Largest diameter for virus-containing droplets | 100 μm as the baseline value  50–200 μm for sensitivity analyses | Assumed [5] |
| $p$ | Pulmonary ventilation rate | 0.48$m^{3}/hr$ | [40] |
| $f_{c}$ | Frequency of cough | 12/hr | [10] |
| $t_{s}$ | Temperature of supply airflow | 14.3°C | [3] |
| $N_{c}$ | Number of droplets generated per cough | 2,000 | Estimated [11] |
| $N_{ip}$ | Number of index patients | 1 | [41] |
| $N_{h}$ | Number of health assistants | 1 | Assumed |
| $N_{np}$ | Number of normal patients at one time point | 38 | Assumed [4] |
| $N_{v}$ | Number of visitors | 0–3 for each patient | Assumed |
| $N_{d}$ | Number of doctors | 4 | Assumed |
| $N_{m}$ | Number of medical students | 19 | [38] |
| $N_{n}$ | Number of nurses | 4 | Assumed [42] |
| $N_{cs}$ | Number of cleaning staff | 1 | Assumed |
| $\eta_{c}$ | Surface cleaning efficiency | 80% | Assumed |
| $P_{w}$ | The probability of nurses washing hands after contacting a patient | 67.3% | Estimated [43] |
| $\eta_{w}$ | Hand washing efficiency | 0.4 | Estimated [44] |

**SI C. Supplemental figures**

**C1 Comparison of aerosol distributions of CFD simulations and multi-zone methods**

**
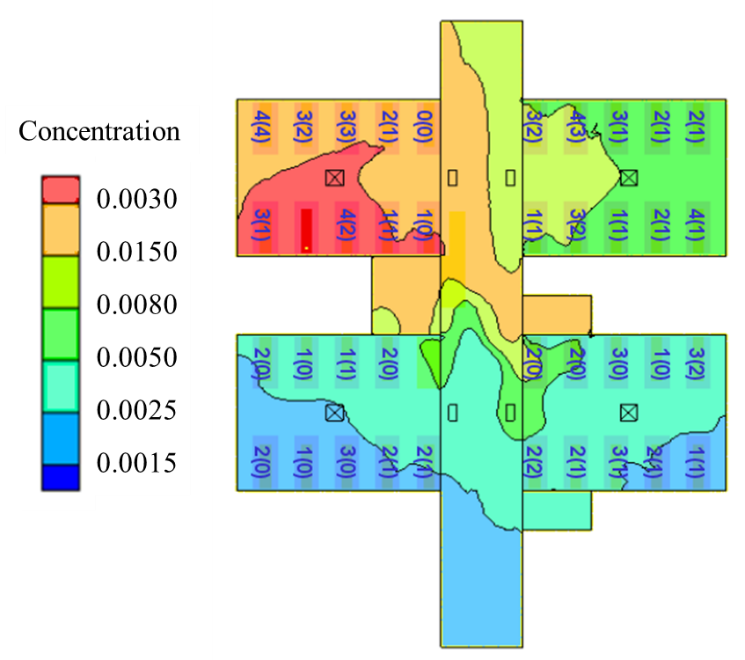

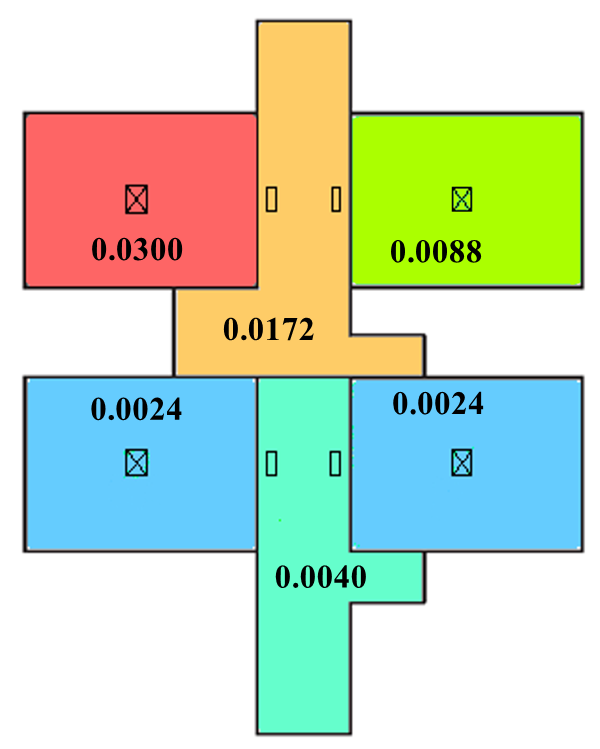
**

**Figure C.** Comparison of normalized aerosol distributions with CFD simulations [4] and multi-zone methods.

**C2 Spatial characteristics of the predicted infection risk patterns (Patterns 2, 4 and 6)**


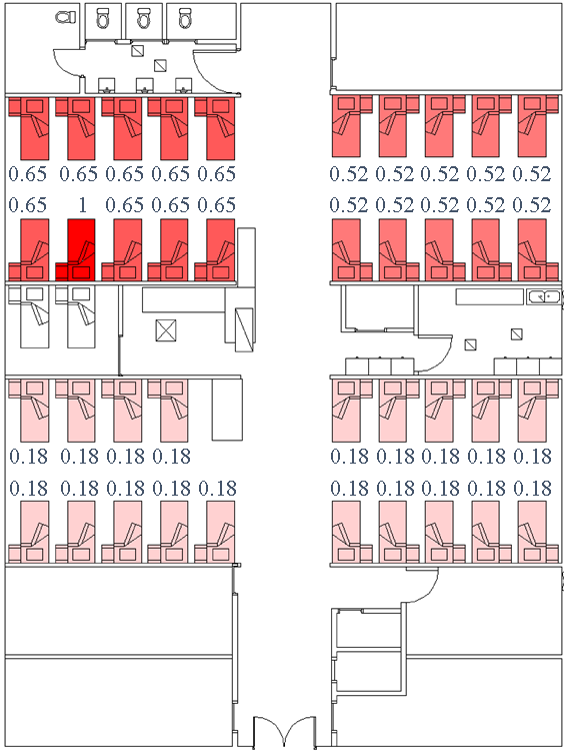

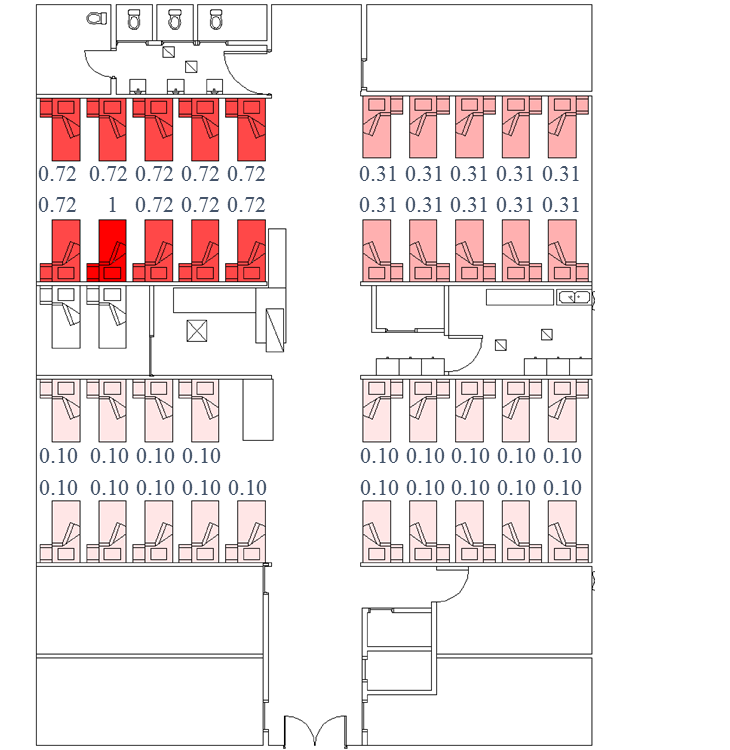


(i) (ii)


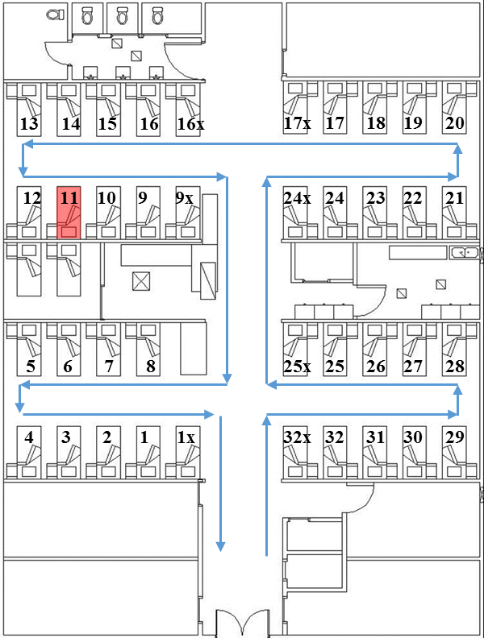

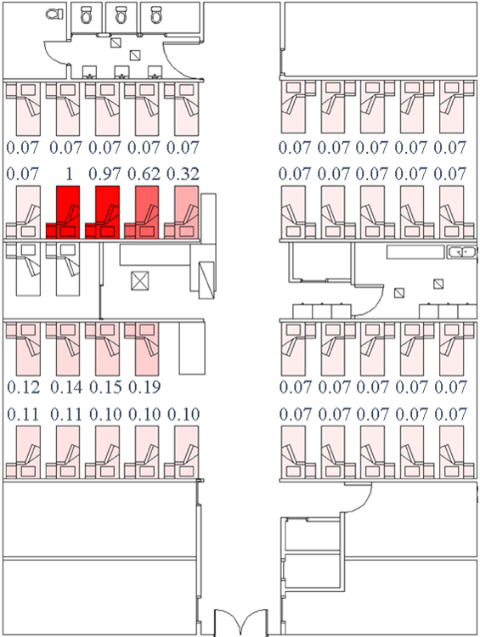


(iii) (iv)


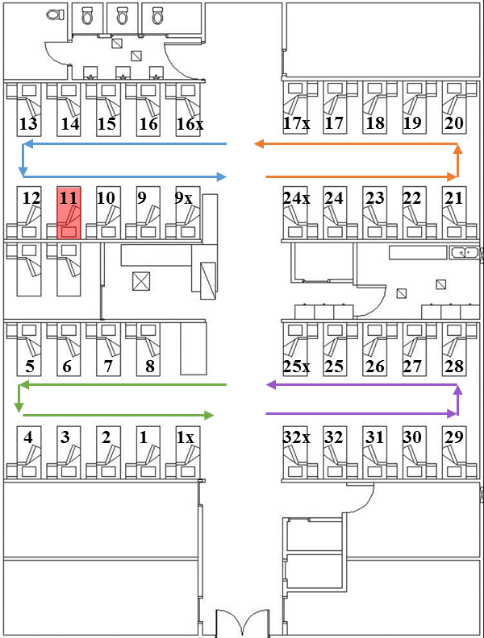

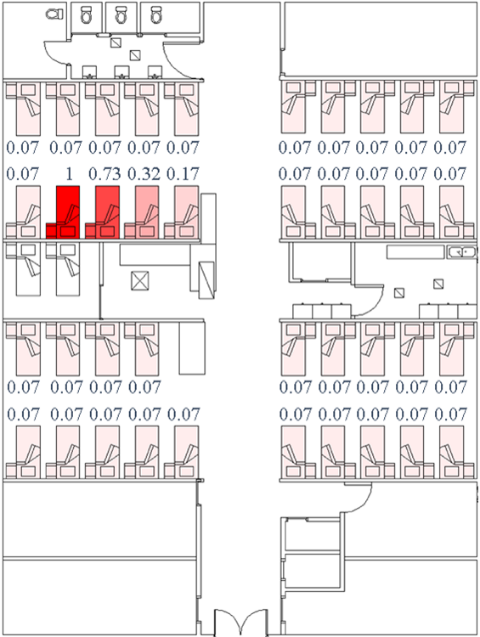


(v) (vi)


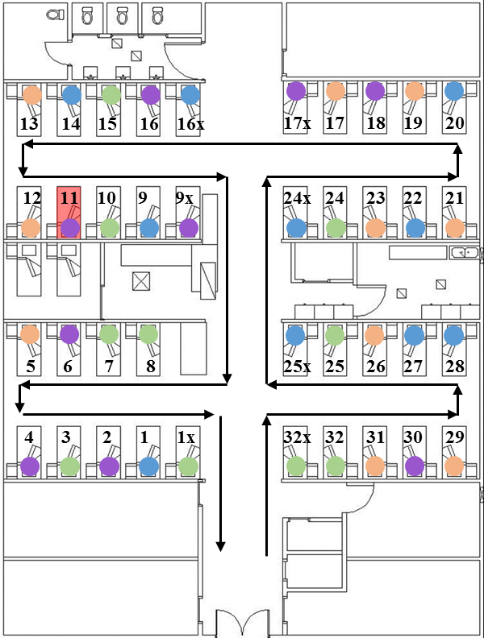

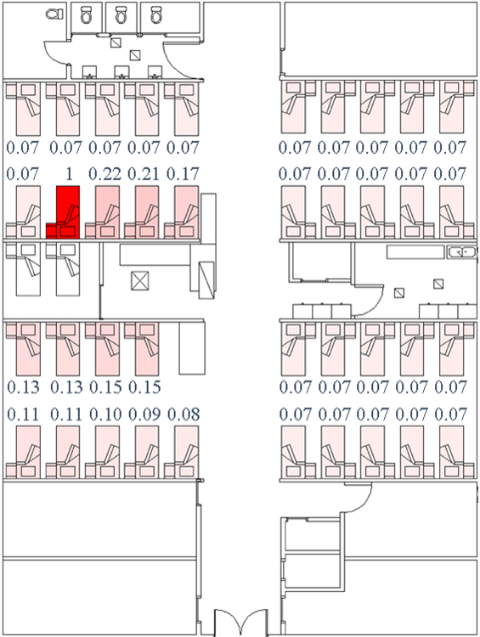


(vii) (viii)


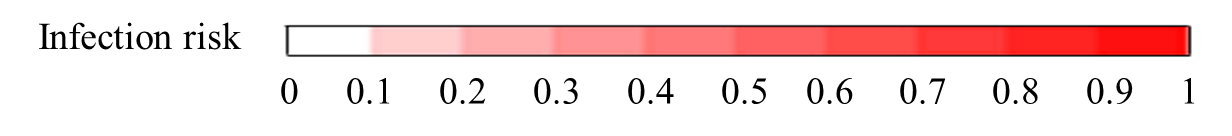


**Figure D.** (i) Reported attack rates distribution [4]. (ii) Predicted average infection risk distribution (for 1,000 simulations) via the long-range airborne route at 24:00 on March 12, the end of the computational period. (iii) HCWs’ routine round Pattern 2. (iv) Predicted average infection risk distribution via the fomite route (Pattern 2). (v) HCWs’ routine round Pattern 4. (vi) Predicted average infection risk distribution via the fomite route (Pattern 4). (vii) HCWs’ routine round Pattern 6. (viii) Predicted average infection risk distribution via the fomite route (Pattern 6). The largest virus-containing droplet size $d_{g}$ = 100 μm, dose–response parameters in respiratory tracts $\eta_{r}$ = 3.2/mRNA copy and on mucous membranes $\eta_{m}$ = 3.2 × 10^-3^/mRNA copy, and the viral load coefficient $c_{L}$ = 10. Bed numbers are marked in black in (iii), (v) and (vii). Reported attack rate and predicted average infection risk for every inpatient are marked in blue in (i), (ii), (iv), (vi) and (viii), respectively. As shown in the legend, the intensity of red shading represents levels of attack rate or infection risk.

**References**

1. Li Y, Delsante A, Symons J. Prediction of natural ventilation in buildings with large openings. Build Environ. 2000;35:191-206.
2. Li Y, Duan S, Yu I, Wong T. Multi-zone modeling of probable SARS virus transmission by airflow between flats in Block E, Amoy Gardens. Indoor air. 2005;15:96-111.
3. Chen C, Zhao B, Yang X, Li Y. Role of two-way airflow owing to temperature difference in severe acute respiratory syndrome transmission: revisiting the largest nosocomial severe acute respiratory syndrome outbreak in Hong Kong. ‎J R Soc Interface. 2011;8:699-710.
4. Li Y, Huang X, Yu IT, Wong TW, Qian H. Role of air distribution in SARS transmission during the largest nosocomial outbreak in Hong Kong. Indoor air. 2005;15:83-95.
5. Gao X. Relative effectiveness of ventilation in community indoor environmentsfor controlling infection. Doctoral dissertations. The University of Hong Kong. 2011. Available from: <https://hub.hku.hk/bitstream/10722/174458/1/FullText.pdf>.
6. ICRP. Human respiratory tract model for radiological protection, a report of task group of the International Commission on Radiological Protection. New York: Elsevier; 1994.
7. Xie X, Li Y, Chwang ATY, et al. How far droplets can move in indoor environments–revisiting the Wells evaporation–falling curve. Indoor air. 2007; 17: 211-225.
8. Liu L, Wei J, Li Y, Ooi A. Evaporation and dispersion of respiratory droplets from coughing. Indoor Air. 2016; doi:10.1111/ina.12297.
9. Xie X, Li Y, Zhang T, Fang HH. Bacterial survival in evaporating deposited droplets on a teflon-coated surface. Appl Microbiol Biotechnol. 2006; 73: 703-712.
10. Nicas M, Jones RM. Relative contributions of four exposure pathways to influenza infection risk. Risk Anal. 2009;29:1292-1303.
11. Chao C, Wan MP, Morawska L, Johnson GR, Ristovski Z, Hargreaves M, et al. Characterization of expiration air jets and droplet size distributions immediately at the mouth opening. J Aerosol Sci. 2009;40:122-133.
12. Atkinson MP, Wein LM. Quantifying the routes of transmission for pandemic influenza. Bull Math Biol. 2008;70:820-867.
13. Thatcher TL, Lai ACK, Moreno-Jackson R, et al. Effects of room furnishings and air speed on particle deposition rates indoors. Atmos Environ. 2002; 36: 1811-1819.
14. Jones RM, Brosseau LM. Aerosol transmission of infectious disease. J Occup Environ Med. 2015; 57: 501-508.
15. Plipat N, Spicknall IH, Koopman JS, Eisenberg JNS. The dynamics of methicillin-resistant Staphylococcus aureus exposure in a hospital model and the potential for environmental intervention. BMC Infect Dis. 2013; 13.
16. Kemeny JG, Snell JL. Finite Markov chains. Princeton, NJ: van Nostrand; 1960.
17. Tikuisis P, Meunier P, Jubenville C. Human body surface area: measurement and prediction using three dimensional body scans. Eur J Appl Physiol. 2001;85:264-271.
18. Lee JY, Choi JW, Kim H. Determination of hand surface area by sex and body shape using alginate. J Physiol Anthropol. 2007;26:475-483.
19. Auyeung W, Canales RA, Leckie JO. The fraction of total hand surface area involved in young children’s outdoor hand-to-object contacts. Environ Res. 2008;108:294-299.
20. Wiertlewski M, Hayward V. Mechanical behaviour of the fingertip in the range of frequencies and displacements relevant to touch. J Biomech. 2012;45:1869-1874.
21. Mackintosh C, Hoffman P. An extended model for transfer of micro-organisms via the hands: differences between organisms and the effect of alcohol disinfection. J Hyg. 1984;92:345-355.
22. Lopez GU. Transfer of microorganisms from fomites to hands and risk assessment of contaminated and disinfected surfaces. Tucson, Arizona: University of Arizona; 2013.
23. Rusin P, Maxwell S, Gerba C. Comparative surface-to-hand and fingertip-to-mouth transfer efficiency of gram-positive bacteria, gram-negative bacteria, and phage. J. Appl. Microbiol. 2002;93:585-592.
24. Lopez GU, Gerba CP, Tamimi AH, Kitajima M, Maxwell SL, Rose JB. Transfer efficiency of bacteria and viruses from porous and nonporous fomites to fingers under different relative humidity conditions. Appl Environ Microbiol. 2013;79:5728-5734.
25. Ijaz M, Brunner A, Sattar S, Nair RC, Johnson-Lussenburg C. Survival characteristics of airborne human coronavirus 229E. J Gen Virol. 1985;66:2743-2748.
26. Wolff MH, Sattar SA, Adegbunrin O, Tetro J. Environmental survival and microbicide inactivation of coronaviruses. Coronaviruses with special emphasis on first insights concerning SARS. Birkhäuser Advances in Infectious Diseases BAID. 2005;201-212.
27. Rabenau H, Cinatl J, Morgenstern B, Bauer G, Preiser W, Doerr H. Stability and inactivation of SARS coronavirus. ‎Med Microbiol Immun. 2005;194:1-6.
28. Chan K, Peiris J, Lam S, Poon L, Yuen K, Seto W. The effects of temperature and relative humidity on the viability of the SARS coronavirus. Adv Virol. 2011; doi:10.1155/2011/734690.
29. Duan SM, Zhao XS, Wen RF, Huang JJ, Pi GH, Zhang SX, et al. Stability of SARS coronavirus in human specimens and environment and its sensitivity to heating and UV irradiation. Biomed Environ Sci. 2003;16:246-255.
30. Peiris J, Chu C, Cheng V, Chan K, Hung I, Poon L, et al. Clinical progression and viral load in a community outbreak of coronavirus-associated SARS pneumonia: A prospective study. Lancet. 2003;361:1767-1772.
31. Legislative Council Select Committee. Outbreak at the Prince of Wales Hospital. 2003. Available from: <http://www.legco.gov.hk/yr03-04/english/sc/sc_sars/reports/ch6.pdf>. Cited 10 Jan 2017.
32. Gryphon Scientific. Supplemental Information: Dose Response Parameters for Gain of Function Pathogens. 2015. Available from: <http://www.gryphonscientific.com/wp-content/uploads/2015/12/Supplemental-Info-Dose-Response.pdf>. Cited 15 Dec 2016.
33. Kwok YLA, Gralton J, Mclaws ML. Face touching: A frequent habit that has implications for hand hygiene. Am J Infect Control. 2015;43:112-114.
34. Elder NC, Sawyer W, Pallerla H, Khaja S, Blacker M. Hand hygiene and face touching in family medicine offices: a Cincinnati Area Research and Improvement Group (CARInG) network study. J Am Board Fam Med. 2014;27:339-346.
35. Hamblin J. How much water do people drink? The Atlantic. 12 Mar 2013. Available from: http://www.theatlantic.com/health/archive/2013/03/how-much-water-do-people-drink/273936/. Cited 15 Dec 2016.
36. Smith J. Am I peeing too much? How to tell what’s normal from frequency to annoying late-night urges, a doctor answers all our pressing questions about going no. 1. Health. 19 Apr 2016. Available from: http://www.health.com/mind-body/am-i-peeing-too-much. Cited 22 Sep 2016.
37. Wanjek C. The poop on pooping: 5 misconceptions explained. Live science. 22 Apr 2014. Available from: http://www.livescience.com/45017-poop-health-misconceptions-truth.html. Cited 12 Sep 2016.
38. Wong TW, Lee CK, Tam W, Lau JTF, Yu TS, Lui SF, et al. Cluster of SARS among medical students exposed to single patient, Hong Kong. Emerg Infect Dis. 2004;10:269-276.
39. Nicas M, Nazaroff WW, Hubbard A. Toward understanding the risk of secondary airborne infection: emission of respirable pathogens. J Occup Environ Hyg. 2005;2:143-154.
40. Chen SC, Chang CF, Liao CM. Predictive models of control strategies involved in containing indoor airborne infections. Indoor Air. 2006;16:469-481.
41. Wong RS, Hui DS. Index patient and SARS outbreak in Hong Kong. Emerg Infect Dis. 2004;10:339-341.
42. Spetz J, Donaldson N, Aydin C, Brown DS. How many nurses per patient? Measurements of nurse staffing in health services research. Health Serv Res. 2008;43:1674-1692.
43. Chau JPC, Thompson DR, Twinn S, Lee DT, Pang SW. An evaluation of hospital hand hygiene practice and glove use in Hong Kong. J Clin Nurs. 2011;20:1319-1328.
44. Pittet D, Dharan S, Touveneau S, Sauvan V, Perneger TV. Bacterial contamination of the hands of hospital staff during routine patient care. Arch Intern Med. 1999;159:821-826.
